# A double blind clinical trial evaluating the relative pharmacokinetics and bioavailability of oral creatine monohydrate when combined with either isomaltulose or dextrose in healthy adult males

**DOI:** 10.1186/1550-2783-9-S1-P14

**Published:** 2012-11-19

**Authors:** Douglas S Kalman, Samantha Feldman, Howard I Schwartz, Diane R Krieger

**Affiliations:** 1 Miami Research Associates. 6141 Sunset Drive Suite 301, Miami, FL. 33143, USA

## Background

Isomaltulose (6-0-α-D-glucopyranosyl-D-fructose) is a low-glycemic, low-insulinemic disaccharide that is absorbed more slowly than conventional sugars (monosaccharides). In sports nutrition, creatine monohydrate is often combined with dextrose (a monosaccharide) for the purpose of enhanced absorption and cellular uptake.

## Methods

In a prospective, randomized, double blind, active-comparator-controlled, parallel group pilot study, 30 male subjects, age 27.0 ± 4.6 years, with BMI of 24.75 ± 1.99 kg/m^2^ and a body surface area (BSA) of 1.953 ± 00.75 m^2^, were randomly assigned to ingest 3 grams of creatine monohydrate (CM) in combination with isomaltulose (ISO) or dextrose (DEX) in 1 of 3 concentrations (5 gm liquid, 17 gm capsules or 50 gm liquid). Rate of absorption (t_Max_) and overall absorption (from BSA adjusted AUC_0-8h_ and C_Max_) of CM was determined via changes in serum creatine over an 8-hour test period. Blood was collected at baseline and 0.5, 1, 2.5, 4 and 8 hours post ingestion with efficacy endpoints including C_Max_, t_Max_, AUC_0-8h_ and λ_Elim_ derived from normalized concentration vs. time curves for serum creatine (AUC by trapezoidal integration). Serum creatine levels were normalized by BSA using the Mosteller formula. For PK parameters, paired Student t test (or Wilcoxon if non-normally distributed) was used and for categorical variables, Fisher Exact test (or Chi-Square if necessary) was used. Statistics were calculated by *R* v2.14.0 (www.r-project.org).

## Results

For the 17 gm concentrations, ISO had a significantly higher C_Max_ than DEX (18.1 ± 1.5 vs 12 ± 1.6 mg/dl*m^2^; p<0.001) and for the 50 gm concentrations, the C_Max_ trended higher for ISO than DEX (19.1 ± 6.4 vs 13.1 ± 3.3 mg/dl*m^2^; p=0.099). The AUC for the 50 gm concentration was significantly higher for ISO than DEX (54.6 ± 9.2 vs 40.3 ± 10; p=0.046). The 17 gm (1.9 ± 0.8 hrs) and 50 gm (1.3 ± 0.7 hrs) concentrations were associated with larger t_Max,_ which trended toward significance over the 5 gm concentration (1 ± 0 hrs) for ISO (p=0.078) and was not significant for DEX. For all 3 concentrations, the C_Max_ and AUC were significantly higher for ISO than DEX (17.8 ± 4.7 vs 13.5 ± 2.8 mg/dl*m^2^ and 50.8 ± 17.1 vs 38.8 ± 10.3; p=0.005 and p=0.027 respectively).

## Conclusions

CM appears to be absorbed more efficiently when combined with ISO over DEX supported by a significantly higher C_max_ for the 17 g concentration and a significantly higher AUC for the 50 g concentration. The 17 and 50 gm formulations appear to be superior to the 5 gm concentration. ISO appears to be a beneficial carbohydrate for facilitating the delivery of creatine to the body.

